# Can vocational advice be delivered in primary care? The Work And Vocational advicE (WAVE) mixed method single arm feasibility study

**DOI:** 10.1136/bmjopen-2025-098768

**Published:** 2025-12-29

**Authors:** Gwenllian Wynne-Jones, Gail Sowden, Ira Madan, Karen Walker-Bone, Carolyn Chew-Graham, Benjamin Saunders, Martyn Lewis, Kieran Bromley, Sue Jowett, Vaughan Parsons, Gemma Mansell, Kendra Cooke, Sarah A Lawton, Catherine Linaker, John Pemberton, Cyrus Cooper, Nadine E Foster

**Affiliations:** 1 School of Medicine, Keele University Faculty of Medicine & Health Sciences, Keele, UK; 2 Connect Health Group Limited, Newcastle upon Tyne, UK; 3 Occupational Health, Safety and Wellbeing Service, Guys and St Thomas NHS Foundation Trust, London, UK; 4 School of Life Sciences and Medicine, King’s College London, London, UK; 5 Monash Centre for Occupational & Environmental Health, Monash University School of Public Health and Preventive Medicine, Melbourne, Victoria, Australia; 6 Clinical Trials Unit, Keele University, Newcastle-under-Lyme, UK; 7 Keele University Faculty of Medicine & Health Sciences, Keele, UK; 8 Health Economics Unit, University of Birmingham, Birmingham, UK; 9 School of Psychology, College of Health and Life Sciences, Aston University, Birmingham, UK; 10 MRC Lifecourse Epidemiology Centre and Centre for Musculoskeletal Health and Work, University of Southampton, Southampton, UK; 11 Research User Group, Impact Accelerator Unit, Keele University, Newcastle-under-Lyme, UK; 12 MRC Lifecourse Epidemiology Unit, University of Southampton and Southampton University Hospitals NHS Trust, Southampton, UK; 13 STARS Education and Research Alliance, Surgical Treatment and Rehabilitation Service, The University of Queensland and Metro North Health, Brisbane, Queensland, Australia

**Keywords:** Primary Care, Occupational Health Services, Psychosocial Intervention, Feasibility Studies

## Abstract

**Objectives:**

Most patients with health conditions necessitating time off work consult in primary care. Offering vocational advice (VA) early within this setting may help them to return to work and reduce sickness absence. Previous research shows the benefits of VA interventions for musculoskeletal pain in primary care, but an intervention for a much broader primary care patient population has yet to be tested. The Work And Vocational advicE feasibility study tested patient identification and recruitment methods, explored participants’ experiences of being invited to the study and their experiences of receiving VA.

**Design:**

A mixed method, single arm feasibility study comprising both quantitative and qualitative analysis of recruitment and participation in the study.

**Setting:**

Primary care.

**Methods:**

The study included participant follow-up by fortnightly Short Message Service text and 6-week questionnaire. Stop/go criteria focus on recruitment and intervention engagement. The semistructured interviews explored participants’ experiences of recruitment and receipt and engagement with the intervention.

**Results:**

19 participants were recruited (4.3% response rate). Identification of participants via retrospective fit-note searches was reasonably successful (13/19 (68%) identified), recruitment stop/go criteria were met with ≥50% of those eligible and expressing an interest recruited. The stop/go criterion for intervention engagement was met with 16/19 (86%) participants having at least one contact with a vocational support worker. Five participants were interviewed; they reported positive experiences of recruitment and felt the VA intervention was acceptable.

**Conclusion:**

This study demonstrates that delivering VA in primary care is feasible and acceptable. To ensure a future trial is feasible, recruitment strategies and data collection methods require additional refinement.

**Trial registration number:**

NCT04543097.

**STRENGTHS AND LIMITATIONS OF THIS STUDY**

- This is the first study to test the feasibility of delivering a vocational advice (VA) intervention to patients who present in primary care with a range of health conditions.
- The study used mixed method to fully explore the feasibility of the delivery of a full trial.
- Recruitment was challenging, indicating that changes in the methods were required before proceeding to a full trial.
- Changes in how sickness absence is managed in primary care due to the COVID-19 pandemic mean that changes in methodology for studies using fit notes as a recruitment point are required.
- The findings can usefully inform the development of the methods for a future trial to ensure that it meets the needs of participants in supporting them to return to work after a period of absence.

## Background

 Absence from work is increasing across European countries, each of which has its own models for managing this absence.^1^ The availability and provision of vocational advice (VA) in the UK is variable and often only accessible to those working for larger organisations.^2^ It is estimated that just 45% of all employees in the UK have access to occupational health (defined as the clinical specialty concerned with prevention and treatment of occupational disease and ill health),^3^ which is lower than comparable countries.^4^ For example, in France, Germany and the Netherlands, all employers must provide occupational health services.^5^ While the majority of healthcare in the UK is delivered through the National Health Service and is free at the point of contact, this service does not provide occupational health.^6^ The Office for National Statistics (ONS) reported that 2.5 million people are reporting long-term sickness as the reason for not being in work; this has risen by 500 000 since 2019 and is not thought to be solely related to the COVID-19 pandemic.^7^ Of concern, long-term sickness absence is rising fastest in younger age groups (aged 25–34 years), meaning that individuals potentially have an extended or even permanent period of economic inactivity due to sickness. Furthermore, the ONS reported that just 16% of those on long-term sickness absence returned to employment between 2021–2022.^7^ Analyses of new claimants applying for the UK unemployment benefit for sick and disabled individuals (Employment and Support Allowance) found that 61% of claimants had sickness absence from their last job and 75% had decided to stop working altogether.^8^ High-quality, timely, VA and support in primary care may improve these outcomes,^9^ improve patients’ health and quality of life and benefit wider society by supporting active engagement in the workforce.^10^ In the UK, sickness absence is managed in primary care through the fit note, historically only provided by a doctor (usually the general practitioners (GPs)), which is required to access sickness absence benefit or occupational sick pay after 7 days of work absence.^11^ While GPs have been expected to have an active role in advising and supporting patients back into work, a role for other health professionals and non-health professionals in managing this interface is recommended.^12 13^ Reforms to UK legislation introduced in 2022 now authorise other clinicians (nurses, physiotherapists, occupational therapists and pharmacists) to issue and certify fit notes (previously referred to as the sick note).^14^ However, fit notes are often issued via an online request and many patients receive a fit note without speaking to a clinician about their health and work.^15^

It is important that people are directed to timely, evidence-based sources of support and advice about their health and work issues (eg, to address obstacles to return to work (RTW)) before they get to the stage where they become long-term absent from the workforce. The UK Government’s report ‘Health is everyone’s business’ highlights the need for improved VA and support to be offered as part of economic recovery plans.^16^ The report focuses on the need to develop and deliver new occupational health models and make greater use of technology to support small and medium enterprises to access occupational health services. The Work And Vocational advicE (WAVE) study aimed to address these challenges by determining the feasibility of a new model of delivery of VA (to support occupational health) via telephone and videoconference, addressing the need for a flexible and technology-based delivery.

This feasibility study is reported in line with the Consolidated Standards of Reporting Trials extension for feasibility and pilot trials.^17^

## Objectives

1. To test patient identification methods, approach to screening for eligibility and recruitment and people’s willingness to engage with a VA intervention in a single group feasibility study.
2. To test data collection processes for response rates and completeness of data.
3. To understand participants’ experiences of being invited to the study, the delivery of the VA intervention and the usefulness of the intervention in supporting them to RTW through semistructured interviews reported in this paper and consultation recordings (published elsewhere).^18^

## Methods

The protocol for the feasibility study is published in full on the National Institute for Health and Care Research (NIHR) journals library.^19^

### Patient and public involvement and engagement

Patients and the public were involved in the development of the research question, participated as a co-applicant on the grant application and at all stages of the design and delivery of the feasibility study. Members with lived experience of work and health supported the development and design of the study methods, reviewed all participant-facing materials, sat on the trial management group, the steering committee and data monitoring committees. People with lived experience also supported the team in interpreting and understanding the results, with one of our members also a co-author on all outputs from the WAVE study.

### Design

A mixed method, single arm feasibility study comprising both quantitative and qualitative analysis was undertaken. Stop/go criteria were used to assist decision making about whether to proceed to a full trial, and linked semistructured interviews were also undertaken.

### Setting

The study screened and recruited participants in primary care with recruitment taking place in general practices in three regions in England: Staffordshire, Wessex and South London.

### Description of the intervention

The intervention delivered was a work-focused VA intervention remotely delivered by vocational support workers (VSWs). The development of the intervention and the training package for VSWs forms the focus of a separate publication currently in progress. In summary, the intervention was based on a logic model detailing key treatment targets (obstacles to RTW) including personal factors: health; cognitions; behaviours; emotions and occupational factors including workplace contact; communication and workplace adjustments. Intervention processes focused on supporting participants to tackle obstacles to RTW and included methods such as goal setting; problem solving; case management and RTW planning. The intervention was delivered using the principles of stepped care and case management, with the VSWs stepping up the intervention when necessary and taking on a case manager role to support participants’ RTW. The intervention delivery could be tailored to each participant and there was no lower or upper limit on consultation, although it was anticipated that most participants would have one to two consultations with fewer requiring more intensive support, based on previous studies.^20^ A letter was sent to the participants’ general practice informing them of their patients’ participation. On completion of the intervention and where required during the intervention, the participants’ general practice received written communication documenting the VA provided.

### Inclusion and exclusion criteria

Eligibility criteria were informed by subgroup analyses of the previous Study of Work And Pain (SWAP) trial data which suggested that the VA intervention may be more effective in those participants who had at least 2 weeks absence from work.^20^

#### Inclusion criteria

1. Adults aged 18 years and over.
2. Currently in paid employment (full or part time).
3. Currently absent from work for at least two consecutive weeks but not more than six continuous months.
4. Receiving a fit note.
5. Have access to a mobile phone that can receive and respond to Short Message Service (SMS) text messages.
6. Able to read and write English.
7. Able to give full informed consent.
8. Willing to participate.

#### Exclusion criteria

1. Long-term work absence defined as longer than six continuous months.
2. Pregnant or on maternity leave.
3. Patients presenting with signs or symptoms indicative of serious illness requiring urgent medical attention (‘red’ flags).
4. Severe mental health problems (eg, severe depression with risk of self-harm, exacerbation of schizophrenia or bipolar disorder, cognitive impairment or lack of capacity) high vulnerability (eg, palliative stages of illness, recent bereavement, dementia).

### Identification and recruitment of potential participants

To test patient identification methods, approach and eligibility for the WAVE study, several recruitment methods were assessed. Recruitment took place between December 2021 and March 2022. A detailed description of the methods of identification and recruitment is reported in the protocol and is summarised below.^21^

#### Method A: identification through automated health informatics Information Technology (IT) Protocol during ‘real time consultations’

Potentially eligible patients were identified using an automated medical record protocol (a ‘pop-up’) which activated when the clinician completed an electronic fit note (eMED3) *during* a consultation with a patient. This pop-up only triggered if the patient met the inclusion/exclusion criteria. The clinician would then ask the patient for consent to share the patient contact details with Keele Clinical Trials Unit (CTU), who then posted the patient a study pack inviting them to the study.

#### Method B: identification through searches of the general practice medical record after consultation where a pop-up is used to assess eligibility on completion of a fit note

Potentially eligible patients were identified using a pop-up which activated when the clinician completed an eMED3 *after* a consultation with a patient. This pop-up only triggered if the patient met the inclusion/exclusion criteria. Patients identified as being potentially eligible were then sent a study pack from the practice to invite them to the study.

#### Method C: identification through retrospective searches of the general practice medical record for all fit notes

The final method was designed to reduce the interruption to consultations that pop-ups bring. Clinicians issued fit notes as usual and a search of patients who had been issued a fit note within the past 7 days was undertaken, with clinicians screening the lists identified to assess eligibility. Practices then sent a study pack to patients inviting them to the study.

For all recruitment methods, on receipt of the study pack, potential participants were asked whether they were still absent from work because of their health condition. Those that were still absent were asked to complete the consent form and baseline questionnaire and return them to Keele CTU.

### Data collection

To test patient identification methods and approach to screening for eligibility and recruitment, the number and proportion of potentially eligible patients identified, invited and consenting to participate was collected via an audit of each of the recruitment methods. Willingness to engage with the VA intervention, the take-up of the offer of the intervention and the steps of the intervention subsequently engaged with were identified from case report forms which described the intervention delivery for each participant.

Self-reported data were collected via postal questionnaire at baseline and at 6 weeks follow-up and by SMS messaging every 2 weeks for a period of 6 weeks. This data collection allowed the processes to be assessed, and any data collection issues related to completeness of data to be identified and adapted should the study progress to a full trial. The following data were collected:

#### Work absence

Participants were asked to report the total number of days absence due to their health condition in the preceding 6 weeks alongside their current work status. RTW was collected by contacting participants on a fortnightly basis by SMS. Contact was maintained until a sustained RTW was achieved (defined as return to *any work* for at least 4 weeks). RTW was measured via SMS text message using the following questions:

- On a scale of 0–10, where 0 is very poor and 10 is very good, how would you rate your general health over the past 2 weeks?
- Have you returned to work? yes/no.
- If yes, on which date did you return to work, for example, 13 September 2021?

Work interference was measured using the Work Productivity Activity Impairment (WPAI) questionnaire.^22^ Work performance was measured using the Single Item Performance Question.^23^

#### Additional measures

A number of measures corresponding to concepts considered important and included in the logic model underpinning the VA intervention were also measured; the publication describing the development of the logic model is in progress. These are reported in full in the protocol but included^21^:

##### Personal health

Physical health and mental health were measured using the Short Form 12.^24 25^ Depression was measured using the Patient Health Questionnaire 8,^26^ anxiety with the Generalised Anxiety Disorder (GAD) 7^27^ and quality of life was measured using the EuroQol-5 Dimensions, five-level version (EQ-5D-5L).^28^

##### Personal influences

The attitudes and beliefs about work questionnaire, used in a previous randomised controlled trial (RCT), was developed to assess how participants view working with health conditions.^20^ The Return-to-Work Self-Efficacy^29^ questionnaire measured changes in a participant’s confidence to return to work.

##### Personal behaviours

Physical activity level was measured using the General Practice Physical Activity Questionnaire 3.^30^

##### Occupational measures

Occupational measures included work absence in the past 12 months, use of other work support services (provided through health services or the participants’ workplace), current job title and characteristics, perceived global stress at work,^31^ satisfaction with work^32^ and how soon the participants expected to resume their normal job without any limitations.

Lastly, participants reported the main health condition (mental ill-health, musculoskeletal conditions or other condition) that resulted in their work absence and sociodemographic data.

### Engagement with the VA intervention: linked semistructured interviews

To understand participants’ experiences of being invited to the study, the delivery of the VA intervention and the usefulness of the intervention in supporting RTW, all participants recruited to the feasibility study were invited to participate in a semistructured interview. Interviews took place at any point after the baseline questionnaire was completed. On receipt of a completed reply slip, the study team contacted the participant by phone to arrange a suitable date and location for the interview. A consent form was completed prior to each interview, either written if face-to-face or audio-recorded consent where interviews were undertaken by phone or video. Topic guides were used to support the interviews and included questions about the individual’s work absence, the acceptability of participant information about the WAVE study and the VA intervention, their experience and views of the recruitment process and their experiences of the VA intervention delivery, content and usefulness.

Engagement with the VA intervention was further assessed through audio recordings of consultations between participants and VSWs. These data informed the feasibility study and are reported elsewhere.^18^

### Sample size

No formal sample size was calculated for the feasibility study; however, the aim was to recruit up to 30 participants, with approximately 10 from each of the three geographical regions. For the semistructured interviews, these participants were all invited to an interview.

### Analysis

To assist decision making about whether to progress to a full trial, the stop/go criteria in the feasibility phase were:

1. Recruitment uptake: uptake of those eligible and who expressed an interest in the study: <25% (red); 25%–49% (amber) and ≥50% (green).
2. Engagement with the VA intervention: the percentage of patients who had at least one contact with a VSW <40% (red), 40%–65% (amber) and >65% (green).

The stop/go criteria were based on the findings of the SWAP trial, which was undertaken in the same setting and used similar methods to identify participants and offer a VA service.^20^ The SWAP trial indicated that of those offered a VA service, 76% took up this offer, with 81% of this group having at least one contact with a VSW. Anticipating that there would be lower engagement given the broader nature of the population included in WAVE, estimates of recruitment uptake and engagement were lowered.

Descriptive analyses of the key feasibility measures were:

- Number (per cent) of patients who were identified in each identification method.
- Number (per cent) of patients who were eligible and interested in taking part in the study.
- Number (per cent) of patients who consented to participate.
- Number (per cent) of study participants who engaged with each step of the study intervention.
- Completeness of questionnaires at baseline and 6 months and completeness of SMS text messaging responses.

#### Analysis of interviews

The audio-recordings of interviews were transcribed in full and anonymised through replacing names with pseudonyms and removing other potentially identifiable information. Data were analysed through an inductive, exploratory framework using thematic analysis and informed by the constant comparison method, looking for connections within and across interviews and across codes, highlighting data consistencies and variation.^33 34^ While it was intended that data collection and analysis be driven by saturation, defined as ‘informational redundancy’,^35^ the final sample size was restricted to the small number of participants in the feasibility study who returned a reply slip in response to the interview invitation.

Anonymised transcripts were systematically coded on a line-by-line basis by the same qualitative researcher who conducted the interviews (BS), with the aid of the software programme Nvivo V.12,^36^ to identify recurrent concepts inductively. Coding was reflexive and recursive, with codes being revisited considering the findings of subsequent data collection. Three interview transcripts were independently coded by another member of the research team (CCG). Both coders have significant experience in qualitative analysis and brought different disciplinary perspectives to the data (BS, medical sociology; CCG, academic general practice). The aim of independent coding was therefore to understand cross-disciplinary perspectives on the data and, through discussion, to come to an agreement on shared meanings and interpretations.

## Results

### Feasibility criterion

**Figure 1:**
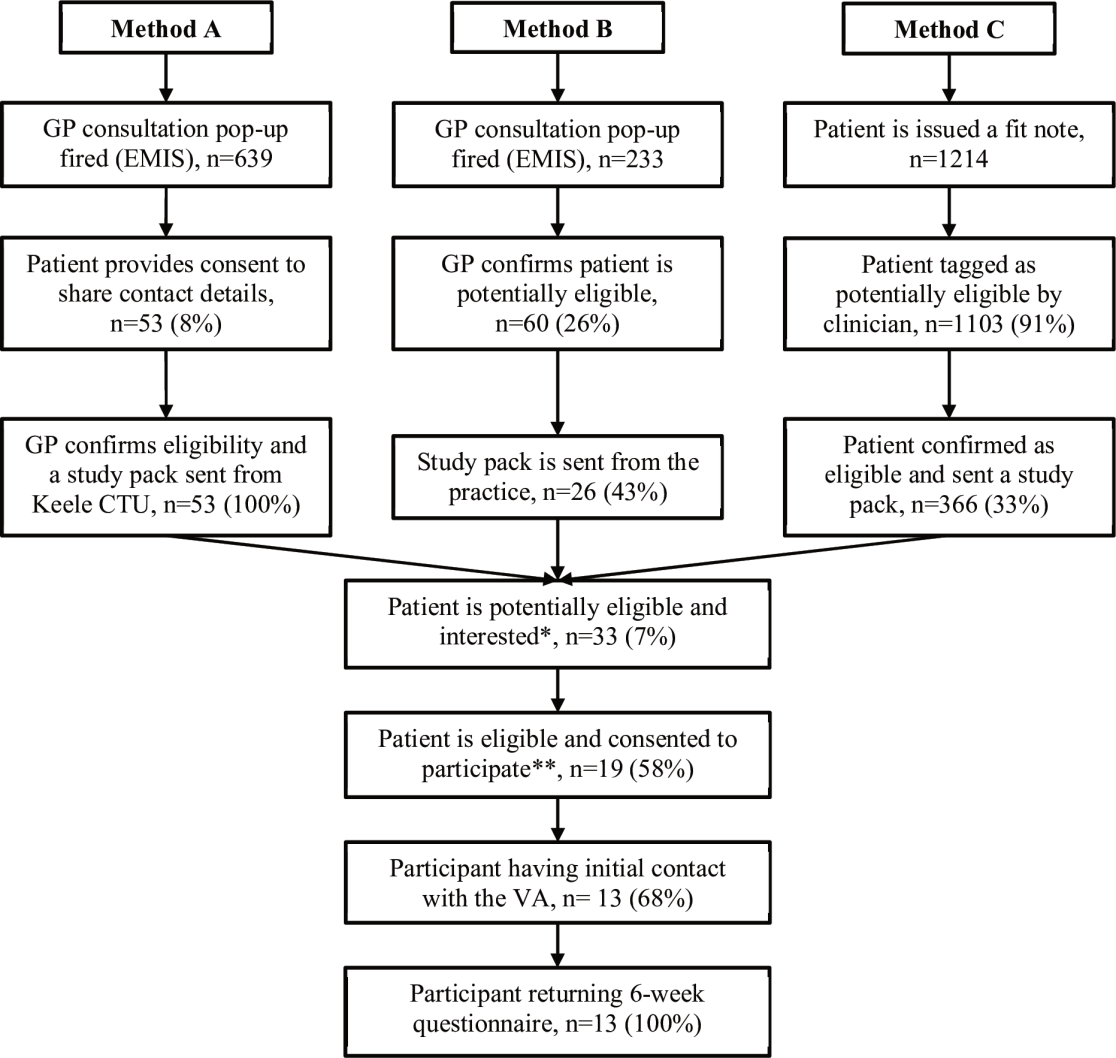
Flow diagram showing the flow of patients throughout the study. *The patient has returned their study pack containing their consent form and/or questionnaire. Potentially eligible refers to the patient being determined as potentially eligible to take part in the study according to the site criteria (ie, fulfilling all inclusion/exclusion criteria at study site assessment). Interested is defined as returned full/partial completion of either or both the baseline questionnaire and/or consent form. **The patient has confirmed in their questionnaire that they are eligible as they have not yet returned to work (‘yes’ response to question A1 confirming that the patient is still absent from work) and provided full consent to participate in the study. CTU, Clinical Trials Unit; GP, general practitioner; VA, vocational advice.

The flow of patients through the feasibility study is shown in figure 1. Across the three methods of patient identification, 445 patients were screened as potentially eligible. Method C (identification through retrospective searches of fit notes in the medical record) was the most successful with 366 (82%) patients identified and mailed a study pack, compared with 53 (12%) in method A (identification through automated searches during real time consultations) and 26 (6%) in method B (identification through searches of the medical record after a consultation where a pop-up is used to assess eligibility).

Of those mailed a study pack, the number of participants potentially eligible and interested, measured through return of either or both their consent form and baseline questionnaire, was low at 33 (7%) across all recruitment methods. Of these, 19 participants (58%) were screened as absent from work, consented and recruited to the feasibility study. This was a green signal for progression to a full trial (ie, >50%) (by stop/go criteria (1)). However, this gave an overall recruitment response rate of 4.3%.

The second feasibility criterion was engagement with the VA intervention measured through the number (%) of study participants who engaged with each step of the WAVE VA intervention. Case report forms, documenting intervention delivery, were available for 14 participants. Of these 14, 13 participants (68%) had initial contact with the VSW, with four (33%) reaching step 1 and eight (76%) reaching step 2 of the intervention; none of the participants reached step 3 (table 1).

**Table 1:** Summary of information recorded in the WAVE Return to work Assessment and action Plan (WRAP) case report form

WRAP	West Midlands (n=7)	Wessex (n=4)	London (n=2)	Total (n=13)	Completeness
Participant contacts					
Total contact made, n (%)					
Phone calls	7 (100%)	4 (100%)	2 (100%)	13 (100%)	13/13 (100%)
Emails sent	5 (71%)	0	2 (100%)	7 (54%)	13/13 (100%)
Video calls	0	0	0	0	13/13 (100%)
Inperson meetings	0	0	0	0	13/13 (100%)
Median (IQR) number of contacts	5 (4, 8)	3.5 (2.5, 5)	9 (8, 10)	5 (3, 8)	13/13 (100%)
Participant was offered but unable to join video calls, n (%)	0	0	0	0	13/13 (100%)
Inperson meeting offered but not possible due to COVID-19, n (%)	7 (100%)	4 (100%)	1 (50%)	12 (92%)	13/13 (100%)
Healthcare professional (HCP) contacts					
Phone calls with HCP, n (%)	0	0	0	0	13/13 (100%)
Wrote to/emailed participant’s HCP, n (%)	1 (14%)	0	0	1 (8%)	13/13 (100%)
Employer contacts					
Total contact made, n (%)	0	0	0	0	13/13 (100%)
In-person meeting offered but not possible due to COVID-19, n (%)	1 (14%)	0	0	1 (8%)	13/13 (100%)
Wrote to/emailed participant’s employer, n (%)	0	0	0	0	13/13 (100%)
Consultation recordings					
First session recorded and verbal consent obtained, n (%)	5 (63%)	2 (50%)	2 (100%)	9 (75%)	13/13 (100%)
Subsequent session recorded and verbal consent obtained, n (%)	3 (38%)	2 (50%)	2 (100%)	7 (58%)	12/13 (92%)
VSW action taken					
Explored participants's current health/work situation, identifying obstacles in returning to work, n (%)	5 (71%)	2 (50%)	2 (100%)	9 (69%)	13/13 (100%)
Provided evidence-based reassurance regarding work and health relationship, n (%)	5 (71%)	2 (50%)	2 (100%)	9 (69%)	13/13 (100%)
Encouraged participants to make contact with their workplace, n (%)	4 (57%)	2 (50%)	1 (50%)	7 (54%)	13/13 (100%)
Discussed problem-solving of perceived obstacles to RTW with participant, n (%)	5 (71%)	2 (50%)	2 (100%)	9 (69%)	13/13 (100%)
Provided information and advice about sleep, n (%)	3 (43%)	1 (25%)	2 (100%)	6 (46%)	13/13 (100%)
Used the techniques of goal setting, n (%)	4 (57%)	2 (50%)	2 (100%)	8 (62%)	13/13 (100%)
Used the techniques of behavioural activation, n (%)	5 (71%)	2 (50%)	1 (50%)	8 (62%)	13/13 (100%)
Used the techniques of action planning, n (%)	5 (71%)	2 (50%)	2 (100%)	9 (69%)	13/13 (100%)
Discussed participant’s RTW plan(s), n (%)	5 (71%)	2 (50%)	1 (50%)	8 (62%)	13/13 (100%)
Signposted to leaflets/resources/other services, n (%)	4 (57%)	1 (25%)	2 (100%)	7 (54%)	13/13 (100%)
Level of intervention delivered, n (%)*					12/14 (86%)*
Step 1	1 (17%)	3 (75%)	0	4 (33%)	
Step 2	5 (83%)	1 (25%)	2 (100%)	8 (67%)	
Step 3	0	0	0	0	
Reason for ending vocational support (VS)					
Participant has achieved 4 weeks sustained RTW, n (%)	1 (14%)	0	1 (50%)	2 (15%)	13/13 (100%)
Participant has decided to terminate employment, n (%)	0	0	0	0	
Employers have terminated the participant’s employment, n (%)	0	0	0	0	
Absent from work for 6 months - signposting advice provided, n (%)	0	1 (25%)	0	1 (8%)	
Absent from work for 6 months - signposting advice not provided, n (%)	0	0	0	0	
Participant no longer wants contact/input from VSW, n (%)	4 (57%)	1 (25%)	0	5 (39%)	
Participant has withdrawn/been withdrawn from the study, n (%)	2 (29%)	2 (50%)	1 (50%)	5 (39%)	
VSW cannot help with the obstacles to RTW, n (%)	0	0	0		
Work outcome					12/13 (92%)
RTW full hours/duties	2 (33%)	2 (50%)	1 (50%)	5 (42%)	
Modified RTW (hours)	0	1 (25%)	0	1 (8%)	
Modified RTW (duties)	1 (17%)	0	0	1 (8%)	
Modified RTW (both hours and duties)	2 (33%)	0	0	2 (17%)	
New job, same employer	0	0	0	0	
Same job, new employer	0	0	0	0	
New job, new employer	0	0	0	0	
Not working in paid employment	0	0	1 (50%)	1 (8%)	
On paid sick leave	1 (17%)	1 (50%)	0	2 (17%)	
On unpaid sick leave	0	0	0	0	
Length of time receiving VS (days), median (IQR)†	43 (40.5, 62.5)	30 (18, 87.3)	37.5 (35.3, 39.8)	41 (33, 43)	13/13 (100%)

* Level of intervention data were available for one additional participant on top of the analysis dataset of n=13, but there were two additional cases of missing data. Hence, % is of n=14.

† The difference in days between date of initial contact and the date that VS ended.

RTW, return to work; VSW, vocational support worker; WAVE, Work And Vocational advicE.

To test feasibility of processes the response rate at 6 months and completeness of data in the questionnaires at baseline and 6 months and the SMS text message data were evaluated.

Overall, there were high levels of completeness for the baseline questionnaire, with the exception of two items on the WPAI questionnaire,^22^ where participants struggled to complete the questions around presenteeism and work productivity (table 2 reports a summary of baseline data with online supplemental table 1 reporting the full dataset). At the 6-week follow-up, 13/19 (68%) participants returned their questionnaire (figure 1). Again, completion of the questionnaire was high with all participants completing questions on measures of absence and work status that are likely to be primary outcomes in a full trial (table 3 reports a summary of 6-week data with online supplemental table 2 reporting the full dataset). Furthermore, the completion of the performance and presenteeism questions of the WPAI^22^ had improved.

**Table 2:** Summary of participant characteristics at baseline

Baseline questionnaire	West Midlands (n=9)	Wessex (n=8)	London (n=2)	Total (n=19)	Data completeness
Age, mean (SD)	54.2 (15.4)	54.6 (10.1)	38.9 (10.4)	52.8 (13.2)	19/19 (100%)
Sex (female), n (%)	6 (67%)	4 (50%)	1 (50%)	11 (58%)	19/19 (100%)
Main health condition causing time off work, n (%)					16/19 (84%)
Aches and pains	2 (29%)	2 (25%)	1 (100%)	5 (31%)	
Mental health problems	5 (71%)	2 (25%)	0	7 (44%)	
Other health condition	0	4 (50%)	0	4 (25%)	
Number of days absence, mean (SD); median (IQR)	14.9 (14.6); 15 (0.8–23.5)	18.4 (12.6); 16 (9.5–26.3)	97 (117.4); 97 (55.5–138.5)	25.6 (40.5); 16 (8.5–25)	18/19 (95%)
Work interference (WPAI:GH), mean (SD)					
Absenteeism	100 (0)	91.4 (22.7)	100 (NA)	96.3 (15)	16/19 (84%)
Presenteeism	100 (0)	90 (NA)	90 (NA)	95 (5.8)	4/19 (21%)
Work productivity	100 (0)	94 (NA)	NA	98 (3.5)	3/19 (16%)
Activity impairment	63.3 (30)	71.3 (23.6)	50 (14.1)	65.3 (25.9)	19/19 (100%)
Work performance (SIPQ), mean (SD)	5.8 (3.8)	3.9 (3.6)	6.0 (4.2)	5.0 (3.7)	19/19 (100%)
SF12-PCS, mean (SD)	36.0 (13.0)	38.0 (15.8)	33.2 (10.8)	36.6 (13.5)	18/19 (95%)
SF12-MCS, mean (SD)	31.1 (10.1)	35.8 (7.6)	40.2 (3.2)	34.2 (8.8)	18/19 (95%)
Depression (PHQ-8), mean (SD)	14.0 (5.3)	8.9 (6.2)	8.0 (7.1)	11.2 (6.1)	19/19 (100%)
Anxiety (GAD-7), mean (SD)	13.1 (4.3)	8.1 (6.4)	9.5 (6.4)	10.6 (5.7)	19/19 (100%)
Attitudes and beliefs to work, mean (SD)	40.1 (4.3)	37.0 (4.2)	42.0 (5.7)	39.0 (4.5)	19/19 (100%)
Return to work self-efficacy (RTW-SE), mean (SD)	3.9 (2.2)	4.8 (2.3)	6.5 (0.9)	4.5 (2.2)	19/19 (100%)
Physical activity level (GPPAQ3), n (%)					17/19 (89%)
Inactive	3 (33%)	1 (17%)	0	4 (24%)	
Moderately inactive	2 (22%)	2 (33%)	1 (50%)	5 (29%)	
Moderately active	2 (22%)	1 (17%)	0	3 (18%)	
Active	2 (22%)	2 (33%)	1 (50%)	5 (29%)	
Working hours, n (%)					18/19 (95%)
Full time (≥35 hours per week)	5 (63%)	6 (75%)	2 (100%)	13 (72%)	
Part time (<35 hours per week)	3 (38%)	2 (25%)	0	5 (28%)	
Satisfaction with work, mean (SD)	5.2 (3.0)	6.1 (2.3)	4 (2.8)	5.5 (2.7)	19/19 (100%)
Health related quality of life (EQ-5D-5L), mean (SD)	0.56 (0.2)	0.56 (0.3)	0.63 (0.05)	0.57 (0.24)	19/19 (100%)

IQR (25%–75%); WPAI:GH (0%–100%), 0%= no impairment/very high productivity, 100%= greatest impairment/very low productivity; SIPQ (0–10), 0= health has not affected work performance, 10= health problems are so bad I am unable to do my job; SF12-PCS (0–100), 0= worst physical health score, 100= best physical health score; SF12-MCS (0–100), 0= worst mental health score, 100= best mental health score; PHQ-8 (0–24), 0= no depression, 24= severe depression; GAD-7 (0–21), 0= no anxiety, 21= severe anxiety; attitudes and beliefs to work (0-60), 0= not impacting return to work, 60= high impact on returning to work; RTW-SE, 19 items (1–10); 1= low self-efficacy, 10= high self-efficacy; EQ-5D-5L (utility) (−0.59 to 1.00), −0.59= worst health utility, 1.00= best health utility.

EQ5D-5L, EuroQol-5 Dimensions, five-level version; GAD, Generalized Anxiety Disorder; GPPAQ, General Practice Physical Activity Questionnaire; PHQ-8, Patient Health Questionnaire Depression Scale; RTW-SE, Return to Work Self-Efficacy; SF12-MCS, Short Form 12 V.2 Mental Component Scale; SF12-PCS, Short Form 12 V.2 Physical Component Scale; SIPQ, Single Item Performance Question; WPAI-GH, Work Productivity and Impairment Questionnaire.

**Table 3:** Summary of 6-week follow-up data

	West Midlands (n=5)	Wessex (n=7)	London (n=1)	Total (n=13)	Completeness
Number of days absence, mean (SD); median (IQR))	22.0 (11.0) 30 (10–30)	20.1 (21.1) 15 (0–42)	0	19.3 (17.3) 15 (0–30)	13/13 (100%)
Current work status, n (%)					13/13 (100%)
Doing your usual job	1 (20%)	4 (57%)	1 (100%)	6 (46%)	
On paid/annual leave	2 (40%)	0	0	2 (15%)	
Working fewer hours	0	1 (14%)	0	1 (8%)	
Doing lighter duties	0	0	0	0	
On paid sick leave	2 (40%)	2 (29%)	0	4 (31%)	
On unpaid sick leave	0	0	0	0	
Work interference (WPAI:GH), mean (SD)					
Absenteeism	78.1 (37.9)	28.6 (48.8)	0 (NA)	39.5 (49)	11/13 (85%)
Presenteeism	40 (14.1)	52 (23.9)	50 (NA)	48.8 (19.6)	8/13 (62%)
Work productivity	83.6 (23)	52 (23.9)	50 (NA)	59.6 (24.9)	8/13 (62%)
Activity impairment	42 (22.8)	52.9 (33.5)	50 (NA)	48.5 (27.6)	13/13 (100%)
Work performance (SIPQ), mean (SD)	9.2 (1.8)	6.4 (3.4)	5 (NA)	7.4 (3.0)	13/13 (100%)
SF12-PCS, mean (SD)	42.7 (11.8)	38.4 (15.9)	41.8 (NA)	40.3 (13.3)	13/13 (100%)
SF12-MCS, mean (SD)	34.8 (10.9)	43.0 (12.0)	38.2 (NA)	39.5 (11.3)	13/13/ (100%)
Depression (PHQ-8), mean (SD)	9.8 (6.4)	8.0 (6.7)	11 (NA)	8.9 (6.1)	13/13 (100%)
Anxiety (GAD-7), mean (SD)	8.0 (5.2)	4.3 (3.5)	14 (NA)	6.5 (4.8)	13/13 (100%)
Attitudes and beliefs to work, mean (SD)	38 (7.2)	36.9 (3.5)	43 (NA)	37.8 (5.1)	13/13 (100%)
Return to work self-efficacy (RTW-SE), mean (SD)	5.6 (2.1)	5.6 (2.5)	7 (NA)	5.7 (2.2)	13/13 (100%)
Working hours, n (%)					11/13 (85%)
Full time (≥35 hours per week)	2 (67%)	5 (71%)	1 (100%)	8 (73%)	
Part time (<35 hours per week)	1 (33%)	2 (29%)	0	3 (27%)	
Satisfaction with work, mean (SD)	6.0 (1.4)	6.0 (1.8)	8 (NA)	6.2 (1.6)	12/13 (92%)
Perceived change in health condition*, n (%)					13/13 (100%)
Completely recovered	0	1 (14%)	0	1 (8%)	
Much improved	2 (40%)	2 (29%)	0	4 (31%)	
Somewhat improved	3 (60%)	3 (43%))	0	6 (46%)	
The same	0	0	1 (100%)	1 (8%)	
Somewhat worse	0	1 (14%)	0	1 (8%)	
Much worse	0	0	0	0	
Health related quality of life (EQ-5D-5L), mean (SD)	0.70 (0.14)	0.70 (0.28)	0.60 (NA)	0.69 (0.22)	13/13 (100%)

WPAI:GH (0%–100%), 0%= no impairment/very high productivity, 100%= greatest impairment/very low productivity; SIPQ (0–10), 0= health has not affected work performance, 10= health problems are so bad I am unable to do my job; SF12-PCS (0–100), 0= worst physical health score, 100= best physical health score; SF12-MCS (0–100), 0= worst mental health score, 100= best mental health score; PHQ-8 (0–24), 0= no depression, 24= severe depression; GAD-7 (0–21), 0= no anxiety, 21= severe anxiety; attitudes and beliefs to work (0–60), 0= not impacting return to work, 60= high impact on returning to work; RTW-SE, 19 items (1–10), 1= low self-efficacy, 10= high self-efficacy; EQ-5D-5L (utility) (−0.59 to 1.00), −0.59= worst health utility, 1.00= best health utility.

* Perceived change in health condition since completing baseline questionnaire.

EQ5D-5L, EuroQol-5 Dimensions, five-level version; GAD-7, Generalized Anxiety Disorder; PHQ-8, Patient Health Questionnaire Depression Scale; RTW-SE, Return to Work Self-Efficacy; SF-12-MCS, Short Form 12 V.2 Mental Component Scale; SF12-PCS, Short Form 12 V.2 Physical Component Scale; SIPQ, Single Item Performance Question; WPAI-GH, Work Productivity and Impairment Questionnaire.

Overall, SMS text message responsiveness was low, with 12/19 (63%) participants responding to the first message at week 2 and 11/19 (58%) at week 4. Subsequent messages had poor response rates, with only 42% (8/19) of participants providing a response to RTW (messages 2 and 3) at week 2 and 32% (6/19) at week 4 (table 4).

**Table 4:** SMS text messaging data

SMS follow-up	West Midlands (n=9)	Wessex (n=8)	London (n=2)	Total (n=19)
Week 2 SMS				
General health*, mean (SD) (n)	6.2 (2.6) (5)	6.3 (37) (6)	5 (NA) (1)	6.2 (3.0) (12)
Have you returned to work? N (%) (n)	0 (0%) (2)	5 (100%) (5)	0 (0%) (1)	5 (63%) (8)
Week 4 SMS				
General health*, mean (SD) (n)	6.2 (1.9) (6)	1.6 (2.0) (5)	0(0)	4.1 (3.0) (11)
Have you returned to work? N (%) (n)	0 (0%) (4)	1 (50%)†(2)	-(0)	1 (17%) (6)
Week 6 SMS				
General health*, mean (SD) (n)	5.5 (0.7) (2)	0 (0)	0 (0)	5.5 (0.7) (2)
Have you returned to work? N (%) (n)	-(0)	-(0)	-(0)	-(0)
Total responses, n (%§)	19/37 (51%)	23/35 (66%)	2/5 (40%)	44/77 (57%)
Total number of participants returning to work, n (%) (n)	0 (0%) (4)	5 (83%) (6)	0 (0%) (1)	5 (46%) (11)
Average time to return to work‡				
Mean (SD)	–	24 (9.9)	–	24 (9.9)
Median (IQR)	–	24 (20.5, 27.5)	–	24 (20.5, 27.5)
Participants responding to 0 texts,¶ n (%)	3 (33%)	2 (25%)	1 (50%)	6 (32%)
Participants responding to 1 text,¶ n (%)	0	0	0	0
Participants responding to 2 texts,¶ n (%)	3 (33%)	0	1 (50%)	4 (21%)
Participants responding to 3+ texts,¶ n (%)	3 (33%)	6 (75%)	0	9 (47%)

* SMS asking the question: ‘On a scale of 0–10, where 0 is very poor and 10 is very good, how would you rate your general health over the past 2 weeks?’.

† Returned to work.

‡ Five participants had provided dates of return to work, however, three were outlier values. Hence, these summary statistics are based on two plausible values.

§ Per cent of all SMS messages sent (both for general health and return to work, with a maximum of three possible responses per time period where a participant has not already returned to work, and two when they currently or previously report returning to work).

¶ Where a response to any text at each time point is included.

SMS, Short Message Service.

### Semistructured interview findings

Analysis of data generated within the interviews explored participants’ experiences of being recruited to and participating in the WAVE feasibility study. Five participants were interviewed. Four participants were female and one male, aged from 45 years to 70 years (mean age: 55), with variation in occupation and reasons for receiving a fit note from their GP (table 5). The length of each interview ranged between 30 min and 63 min (average: 48 min).

**Table 5:** Interview study participant characteristics

Gender	Occupation type	Reason for fit note	No. of VSW consultations at time of interview
Female	Youth work	MSK	Three
Male	Emergency services	MSK	Did not consult with a VSW
Female	Healthcare work	MH	Two
Female	Emergency services	MH	Two
Female	Healthcare work	MH	Two

MH, mental ill-health; MSK, musculoskeletal; VSW, vocational support worker.

Three main themes were identified through the analysis, the results section provides summary of these themes with full extracts reported in online supplemental table 3:

1. Work absence and concerns about return to work.
2. Views towards feasibility study processes.
3. Acceptability and perceived value of the VSW service.

#### Work absence and concerns about return to work

Participants reported concerns around work absence and RTW including workplace stress negatively impacting on health, adaptations to work routines as a result of the COVID-19 pandemic adding additional pressure for some and separation of work and home life being difficult leading to further negative health impacts. Support in the workplace was reported to be variable; some participants expressed concern about not receiving appropriate support on returning, whereas others reported accessing occupational health services at work and being well supported in their RTW planning.

We’ve got a really good Occupational Health department. I’d actually self-referred to them before I went off because of the issues. I sort of self-referred in terms of ‘are there any things that I could or should be doing to make me deal with this better’, and as it turned out I think everyone including the line managers agree that it’s a situational thing. It’s not something about me. So yeah, I have seen occupational health, a counsellor from there. Gosh, probably about six or seven times over the last two or three months. (Female participant, in their 70s)

A common concern was that the participants’ health condition would continue to prevent them fulfilling their job role and that they were unable to consider RTW planning until their health situation had improved. Despite these concerns, all participants expressed a desire to RTW, noting the positive impact work had for them.

Int: How important is it that you can actually return to work in the future to you?

P: Yes, for my sanity really. You know it’s a job I've done since I was sixteen. I only have eight years left before I can retire…and this is not the way I would have wanted to end my career really…it would be a sad way to leave. (Female participant, in their 50s)

#### Theme 2: views towards feasibility study processes

All participants found it acceptable to be invited into the feasibility study. It was reported that first being alerted to the study by receiving a study pack through the post was acceptable, regardless of whether this was sent by their GP surgery or directly from the CTU. Some reported that they would not have felt it appropriate for their GP to explain the study to them in their consultation prior to receiving the study pack, as they would have had difficulty taking the information on board. Participants suggested that the WAVE study and intervention was not only acceptable in the context of the COVID-19 pandemic, but potentially of even greater relevance during this time, given the additional work pressures and shared concerns people were experiencing as a result of the COVID-19 pandemic:

I think [WAVE] is acceptable, definitely, and because we’re in such a stressful time, albeit we seem to be coming out the other end now, but we don’t know where it’s going to go, but it’s good that you can do it when most people are under stress, not just certain people. Most people are under the same sort of stress. (Female participant, in their 50s)

All participants also reported that the patient information and consent form received as part of the study pack were clear, and that based on this information they understood the aims and purpose of WAVE. The baseline questionnaire was reported to be acceptable in its use of language and length of the questionnaire. While participants reported that, in general, they found it straightforward to complete, four participants reported either having difficulty in answering some questions with binary response options or feeling that some of the questions were not applicable to them:

Some of the questions, because I think a lot of it was about physical things, it didn’t sort of directly apply to me, anything like asking ‘do you do heavy lifting?’, and all that sort of thing. So for me I suppose it was a mental health thing more than a physical. A lot of it was easy to fill in because it wasn’t really kind of applicable in a way. (Female participant, in their 50s)

In relation to the receipt of SMS text messages*,* four participants reported being uncertain at the time when the messages were received about who had sent them, due to the sender being displayed only as mobile phone number, without mention of the WAVE study. This uncertainty led to concerns that the text messages may have been sent from participants’ workplaces:

I got sent a couple of texts and I thought ‘oh no this is from occupational health and I’m not going to answer it because I don’t know…’ as it was an unknown number and I thought ‘oh I don’t know this number, I’m not going to just answer these random questions’. (Female participant, in their 40s)

#### Theme 3: acceptability and perceived value of the VSW service

One participant did not recall being contacted by a VSW. The other four participants reported being initially contacted by the VSW via telephone to arrange a suitable time for a consultation, following which all consultations were carried out via telephone. The participants reported finding this method of contact acceptable, particularly in light of social distancing guidance at the time due to COVID-19, and they felt able to effectively build rapport with the VSW via telephone. There was, however, the suggestion by a few of the participants that face-to-face contact could have further improved their experience, making it easier to ‘open up’.

While only one participant who had consulted with the VSW had returned to work at the time of interview, they all reported feeling that the VSW service had been beneficial to them. Consulting with the VSW was seen to have value in several different ways; for instance, participants reported the benefits of interpersonal support provided by the VSW and having someone to talk to about their concerns. Continuity of support and ongoing contact provided by the VSW was also highlighted as valuable.

I found it really useful for talking through some of the things that perhaps at the start of the call I hadn’t actually realised were playing on my mind that much, which was useful and we sort of talked through some things that I could do to try and ease my mind about that really. It is nice being able to talk to somebody about things. (Female participant, in their 70s)

The support provided by VSWs in developing an action plan for RTW was another aspect of the intervention that was considered valuable. However, the timing of RTW discussions was highlighted, with these conversations seen as being of lesser use early in the participant’s work absence, if they did not feel close to considering RTW. While participants who received the VSW service all reported finding it beneficial, it was felt that support from a VSW would be of particular benefit to individuals who are not currently receiving support from their workplace, or do not have access to an occupational health department. Those who already had access to this support reported some overlaps between the VSW service and the support they were already receiving; for instance, where a phased return or workplace adjustments had already been discussed:

She sent me an action plan through, and you know, she said ‘Well okay so if that’s what you’re worried about (the timeframe for returning to full duties), then how can you find that out?’; and she said ‘Could you ask your line manager?’ and I sort of said I could do, but I don’t really want to. I don’t know why, I just didn’t feel entirely comfortable…so what came out of it is that I said, ‘I need to understand really myself exactly what the policy says.’ So yeah, what we’d agreed is I either need to look that up myself or next time I see my counsellor, to go through that properly with her…so yeah, we’d talked through the various ways of how I could sort of put my mind at rest really. (Female participant, in their 40s)

For participants who did not feel that their workplace had provided enough support to them, it was reported that support from the VSW could ‘add weight’ to discussions with work. The VSW had not directly contacted any of the participants’ workplaces, but some did report having discussed this, and all felt that this would be acceptable were it deemed necessary. However, some concerns were expressed about workplaces not being receptive to communication with the VSW, particularly in relation to issues of confidentiality and adherence to General Data Protection Regulations.

## Discussion

The WAVE feasibility study explored the delivery of a VA intervention for adults in primary care certified absent from work for at least 2 weeks. The study experienced significant operational challenges, with the start of recruitment coinciding with the first wave of the COVID-19 vaccine roll-out by general practices and national lockdowns (December 2020–March 2021). Despite this, the study met the two stop/go criteria, recruiting sufficient participants who were eligible and expressed an interest in the study (19/30 (58%)) and sufficient participants engaging with the VA intervention measured as having at least one contact with the VSW (16/19 (84%)). There were, however, some areas that could be refined when planning a full trial. While questionnaire completion was good at both baseline and the 6-weeks follow-up, the use of SMS text messaging to gather shorter-term data on RTW was less successful. Interviews with participants indicated that they were uncertain who had sent the messages as there was no mention of the WAVE study. The SMS method of data collection should be refined in a full trial. Analysis of the qualitative data indicated that while the methods of inviting participants into the study were feasible and acceptable and the study information was clearly understood, recruitment was lower than anticipated. Steps were taken to increase recruitment by introducing automated methods to identify potential participants from the medical record. When planning a full trial, these automated methods would need to be implemented to support recruitment. Lastly, participants reported that telephone contact with the VSW was seen as acceptable and did not pose a barrier to building rapport, indicating that this would also be an appropriate form of contact for future delivery of a VA intervention. Furthermore, this type of intervention was seen as having even greater relevance given additional work pressures during the pandemic.

### Strengths and limitations of the study

This is the first study to test the feasibility of delivering a VA intervention to patients who present in primary care, regardless of their health condition where previous research in this area has included specific health conditions.^20 37 38^ Focusing on one health condition may mean that those with other comorbid conditions are unable to access VA or support is provided in a silo. The WAVE intervention has the potential to address this gap in service provision, given the intervention appears suitable for a wider primary care population. A further strength of the WAVE feasibility study is that it has tested three methods of participant recruitment. The subsequent learning from trialling these methods helped to identify the optimal recruitment strategy for a future trial, that is, one which focuses on automated identification and invitation as far as possible. Additionally, the mixed method approach using the interview data reported here and recordings of intervention delivery reported elsewhere^18^ is a key strength, since it has allowed the examination of not only the recruitment numbers from the different methods tested but also the fidelity of the delivery of the intervention and participants’ experiences of being invited to join the study and engage with the WAVE intervention. These findings can usefully inform the development of the methods for a future trial and also the WAVE intervention itself, to ensure that it meets the needs of participants in supporting them to RTW after a period of absence.

The study was limited by low recruitment (19 of the targeted 30 participants (63%)), and fewer participants agreed to take part in the interviews than was anticipated. This is set within the broader context of when the study was conducted. Our study was constrained by the unprecedented impacts of the COVID-19 pandemic which influenced not only primary care, where the study was delivered, but also the wider context of work where there was a shift in working conditions for many people.^39 40^ The delivery of primary care moved to a predominantly online service, with changes made to many clinical and administrative processes including moving fit notes to an online request system when previously they required consultation with a clinician.^15^

### Comparison with wider literature

The COVID-19 pandemic has resulted in a prolonged period of exceptional pressure on primary care services which have moved to provide care in different ways, including remote consulting.^41^ Research such as the WAVE feasibility study, while impacted by this rapid shift, is still relevant and arguably more so as the recent report, ‘Health is everyone’s business’, highlights the need for improved VA and support to be offered as part of the economic recovery plans.^16^ The report focuses on the need to support new occupational health models and make greater use of technology to support small and medium-sized enterprises to access occupational health services, both of which the WAVE feasibility study delivers.^16^ There are continued concerns over the future availability of a responsive multidisciplinary occupational health workforce.^42^ Government plans to respond to this perceived gap include considering methods to promote the expansion of clinical roles and improving occupational health multidisciplinary workforce models which capture both clinical and non-clinical roles and developing new training and career transition pathways.^16^

It is important to look at the wider literature to set the WAVE feasibility findings in context of not just the policy landscape but also recent studies exploring the provision of VA and particularly the specific issues identified here of recruitment and data collection challenges. Recent reviews of interventions on this topic focus on RTW. Most trials are conducted in European countries and include primarily those with mental health or musculoskeletal conditions. Mixed populations, such as those included in the WAVE feasibility, were not common, and settings were varied but commonly delivered as intensive interventions and in the workplace setting.^43^,^46^ Including a population with varied health conditions and setting the WAVE feasibility study within the primary care setting and early in a person’s work absence is a novel concept and one that may influence progression to long-term absence.

When considering the challenges in delivering trials within health and work, it is important to explore whether there are similarities between the challenges faced in the WAVE feasibility study and other research. Some UK trials have also faced similar challenges in recruitment^47 48^ which are not solely COVID-19 related. Looking at trials from non-UK countries, there is again a challenging picture for recruitment.^49^ While some of these recruitment difficulties are related to COVID-19 in recent studies, there were also significant challenges prior to this, which are related to the lack of a systematic approach to health and work and also to the sensitive nature of conversations around health and work that may discourage participation.^43^,^4548^ The second area identified for refinement in this feasibility study was the use of SMS messages to collect data. Use of SMS messages is not a new concept and has been demonstrated to be a useful method of collecting short term data.^50 51^ Based on the qualitative findings from the WAVE feasibility study, it is the method by which SMS messages are communicated that needs some refinement to ensure participants know it is from the study team.

Conducting research such as the WAVE study supports the development of evidence-based models for provision of VA and support for those without access to occupational health.

### Implications

The WAVE feasibility study highlights the challenges of working within changing clinical (primary care) and social environments (COVID-19). However, the findings align with the UK Government emphasis on improving VA as part of economic recovery plans and as such, further work is warranted in exploring how VA can be successfully integrated into healthcare systems. There are some clear implications arising from this feasibility study. First recruitment to studies focussing on health and work is likely to be challenging, and when planning a future trial, this needs to be considered by extending recruitment periods and reviewing recruitment methods to ensure they are appropriate, in particular, where there is a changing health and employment landscape. Second, when using technology to support data collection, in this case, SMS text messaging, it is important to ensure that it is clear that this is from the trial team. Lack of clarity can lead to suspicions about the origin; getting this right will support crucial short-term data collection.

## Conclusions

The WAVE feasibility study demonstrated that delivering VA in primary care is feasible, participants were successfully identified and recruited to the study and participants engaged in the VA intervention. Assessment of data collection processes indicated that response rates were acceptable, and data completion was very good. Importantly, participants reported that their experience of invitation and recruitment to the study was acceptable and that the VA intervention was useful in supporting them to RTW. The study has identified areas for refinement of recruitment strategies and indicated that clearer communication, when using SMS messages, is required to maximise the potential impact. Progression to a full WAVE trial is indicated taking account of these findings and the implications noted.

## Supplementary material

10.1136/bmjopen-2025-098768 online supplemental file 1

## Data Availability

Data are available upon reasonable request.
