# Supplementary material for: Can vocational advice be delivered in primary care? The Work And Vocational advicE (WAVE) mixed method single arm feasibility study
Source: BMJ Open. 2025 Dec 29;15(12):e098768. doi: 10.1136/bmjopen-2025-098768 (PMC12750802; doi:10.1136/bmjopen-2025-098768)
Supplement: Supplementary file 2 [file bmjopen-2025-098768.reviewer_comments.pdf]

PEER REVIEW HISTORY

BMJ Open publishes all reviews undertaken for accepted manuscripts. Reviewers are asked to complete a checklist review form and are provided with free text boxes to elaborate on their assessment. These free text comments are reproduced below.

ARTICLE DETAILS

Title (Provisional)

Can vocational advice be delivered in primary care? The Work And Vocational advicE (WAVE) mixed method single arm feasibility study (NCT04543097)

Authors

Wynne-Jones, Gwenllian; Sowden, G; Bromley, Kieran; Linaker, Catherine; Pemberton, John; MADAN, IRA; Walker-Bone, Karen; Chew-Graham, Carolyn; Saunders, Benjamin; Lewis, Martyn; Jowett, Sue; Parsons, Vaughan; Mansell, Gemma; Cooke, Kendra; Lawton, Sarah Ann; Cooper, Cyrus; Foster, Nadine E.

VERSION 1 - REVIEW

**Reviewer**	**1**
**Name**	**Dewi, Ayu Putu Madri**
**Affiliation**	**Amsterdam UMC Location AMC, Epidemiology and Data Science**
**Date**	**12-Mar-2025**
**COI**	**None**

I am an Open Science researcher, who has checked your manuscript for its adherence to a number of Open Science practices.

Below you find the checklist that I used to assess your manuscript for adherence to a number of these practices.

Based on this assessment, I would suggest the following refinements/additions:

1. Availability of a protocol: OK

2. Pre-registration: OK

3. Open Materials: OK

4. Open Data: Given the confidentiality of the data, please specify whether de-identified, metadata, or a subset of the data can still be made publicly available.

5. Open Code: Please provide (a link to) the code used for the analyses in this study.

6. Citations: Please provide citations for your data/codes.

7. Software and tools used are described: OK

8. Open access publication: OK

9. Reporting checklist provided: OK

10. Preprint: Please state explicitly whether and where the preprint of this study can be found.

**Reviewer**	**2**
**Name**	**Kwan, Amanda**
**Affiliation**	**The University of British Columbia - Vancouver Campus**
**Date**	**30-Jun-2025**
**COI**	**None**

Interesting work – please address the comments below (separated major vs minor). Thanks!

Major Comments:

- Writing could be clearer and more concise, please edit accordingly. Some parts were difficult to understand due to writing and grammar.

- Background is missing relation to existing research e.g., Where does this fit with existing work? Have other European countries trialed similar approaches? Etc. - please include.

- What is the go/stop criteria based on? Elaborate on decision making

- RESULTS: How many people were eligible and not interested? Do you know why not? Does the recruitment uptake not use this number vs those that were eligible and interested? If the objective is to test people's willingness to engage w VA interventions, should it not consider all those that are eligible and not just those that are interested? Please clarify.

- Does reporting sex instead of gender align w current best practices?

- Concerns around anonymity for reporting such small samples sizes < 5 and especially given participant demographics are provided with interview quotes

- Themes presented are more short summaries of what was said – present as summaries or suggest revision

- Clarify how many VSW consultations are meant to be part of the intervention? Is <2 consultations on average enough for participants to speak to the usefulness?

- Discussion is weak and more a summary of the results. Missing how the results compare to existing literature (e.g., have other studies used and been more successful w SMS text messaging for data collection), and an interpretation of the results/value added, etc.. Suggest revision.

- Implication section is disconnected from study findings and it's not clear how findings can be used to inform future trials or how it is being used to inform this study's next steps. Please elaborate.

- Conclusion states "The study has identified areas for refinement of recruitment strategies …" but these refinements have not been thoroughly presented or discussed.

Minor comments:

- Describe the healthcare system in the UK (e.g., public vs private) and the potential influence of it

- Define/explain occupational health

- Include the average access to occupation health for other similar countries in comparison (i.e., how much lower is access in the UK?)

- How is the study/intervention integrated into primary care practices? Does feasibility depend on clinician buy-in? Please elaborate.

- Intro background: replace "good quality" with "high quality". Also feels like there's a word missing before "active engagement … "

- Provide more context around fit notes in the UK e.g., are they required? Under what circumstances, etc.

- Methods, Design: should be in proper full sentences

- Include ethics coverage in methods

- Suggest moving description of intervention earlier so it's clear what is being talked about

- Add citation for NVivo

- What is the progression criteria? Is this explained somewhere?

- Include when interviews were conducted in relation to surveys and rationale for why only 5 were conducted. Is that representative of the experience?

- Article summary says, "This is the first study to test the feasibility of delivering a VA intervention to patients who present in primary care, regardless of their health condition." But the study excludes people with serious illness and severe mental health. Contradictory, please clarify.

VERSION 1 - AUTHOR RESPONSE

Response to reviewers comments

**Comment from reviewer**	**Response**
Based on this assessment, I would suggest the following refinements/additions:<br>1. Availability of a protocol: OK<br>2. Pre-registration: OK<br>3. Open Materials: OK<br><br>4. Open Data: Given the confidentiality of the data, please specify whether de-identified, metadata, or a subset of the data can still be made publicly available.<br><br>5. Open Code: Please provide (a link to) the code used for the analyses in this study.<br><br>6. Citations: Please provide citations for your data/codes.<br><br>7. Software and tools used are described: OK<br><br>8. Open access publication: OK<br><br>9. Reporting checklist provided: OK<br><br>10. Preprint: Please state explicitly whether and where the preprint of this study can be found.	Many thanks for the review focussed on open data, the advice provided is very helpful and we have made some amendments and plans as detailed below.<br><br>Response to Q4, 5 and 6. Availability of data and analyses code.<br><br>As detailed on page 32 all data are available on request following our institutional process. However, to facilitate open data we are working towards uploading the data to Keele University's Data Repository (Welcome to Keele Data Repository - Keele Data) to enable reproducibility of some of the analyses. We are also in the processes of uploading the code used in the analyses presented in this paper. We acknowledge that some of the data we hold is sensitive and therefore will only be uploaded of it can be anonymised and we are confident in the level of anonymity. Whilst the code is available we are in the process of gaining a reference for this.
Major comments	
Major Comments:<br>- Writing could be clearer and more concise, please edit accordingly. Some parts were difficult to understand due to writing and grammar.	We have reviewed the paper for readability and made some edits thrughout which we hope will address the reviewers concerns.
- Background is missing relation to existing research e.g., Where does this fit with existing work? Have other European countries trialed similar approaches? Etc. - please include.	We have included more information on the provision of occupational health within other European countries; this allows the reader to see where the UK fits in terms of management of occupational health services.<br><br>Discussion of trials in this area is now included in the discussion section "comparison with other literature".
- What is the go/stop criteria based on? Elaborate on decision making	We have updated this section to clarify how decisions on the stop/go criteria were made.

	Essentially the decisions were based on a previous trial in the same setting offering a similar vocational advice service, although we did take account of the differing populations in our estimates.
- RESULTS: How many people were eligible and not interested? Do you know why not? Does the recruitment uptake not use this number vs those that were eligible and interested? If the objective is to test people's willingness to engage w VA interventions, should it not consider all those that are eligible and not just those that are interested? Please clarify.	The closest figure to those who were potentially eligible and not interested can be based on those who were sent a study pack which is 445. However, as participants have not completed the screening questions at the start of the questionnaire, we cannot be certain that they are eligible e.g. having been absent for two weeks. Some may not have reached two weeks absence and others may have returned to work by the time the questionnaire are received.<br><br>The recruitment uptake was based on those potentially eligible who had completed the screening questions, this decision was made between the trial team, the steering committee and the funders to understand the likelihood of recruiting from that specific population rather than those who may not meet the eligibility criteria.<br><br>As the questionnaires of those potentially eligible were not returned, we don't have any information on reasons for non-response.
- Does reporting sex instead of gender align w current best practices?	At the time this feasibility study was set up (2018/19) sex was the current best practice in terms of gathering data. As time has moved on so too has the recording of this data and with hindsight it would have been of value to record both sex and gender.
- Concerns around anonymity for reporting such small samples sizes < 5 and especially given participant demographics are provided with interview quotes	We have amended table 5 and the associated text to address this issue and avoid the potential for a participant to be identified.
- Themes presented are more short summaries of what was said – present as summaries or suggest revision	The qualitative data were analysed using methods of thematic analysis, including line-by-line coding and iterative development of themes (as outlined in the methods section of the paper), and therefore the data are presented under themes. However, the reviewer rightly points out that in the results section we give a summary rather than full articulation of

	these themes. This has been clarified in the manuscript on page 24.
- Clarify how many VSW consultations are meant to be part of the intervention? Is <2 consultations on average enough for participants to speak to the usefulness?	We have included a sentence in the section describing our intervention to clarify this point. Essentially intervention delivery was tailored to each participant with the expectation that most would require one to two consultations with fewer requiring more intensive support.
- Discussion is weak and more a summary of the results. Missing how the results compare to existing literature (e.g., have other studies used and been more successful w SMS text messaging for data collection), and an interpretation of the results/value added, etc.. Suggest revision.	We have amended the discussion extensively included more reference to other studies which have attempted to address a similar topic. We have highlight where these studies align with the WAVE feasibility and where there are difference with particular reference to the challenges of recruitment and data collection.
- Implication section is disconnected from study findings and it's not clear how findings can be used to inform future trials or how it is being used to inform this study's next steps. Please elaborate.	We agree that the implications section is not sufficient and have updated this accordingly to tie it more firmly to the study's recommendations.
- Conclusion states "The study has identified areas for refinement of recruitment strategies …" but these refinements have not been thoroughly presented or discussed.	We have now amended the first paragraph of the discussion to highlight the areas that need to be refined and ensured that these link to the conclusion more firmly.
Minor comments	
- Describe the healthcare system in the UK (e.g., public vs private) and the potential influence of it	We have included the following statement (and a reference) about the UK healthcare system and where occupational health fits within that "The majority of healthcare in the UK is delivered through the National Health Service and is free at the point of contact, however, this service"
- Define/explain occupational health	We have included the following definition of occupational health in the background: "…which can be defined as the clinical specialty concerned with prevention and treatment of occupational disease and il health" and referenced this as appropriate.
- Include the average access to occupation health for other similar countries in comparison (i.e., how much lower is access in the UK?)	We have expanded on the first paragraph of the background to indicate some of the countries where occupational health services are more accessible and included an additional reference to support this.
- How is the study/intervention integrated into primary care practices? Does feasibility depend on clinician buy-in? Please	We have updated the section on the intervention to include a brief statement of how the VSW integrated the service with

elaborate.	primary care "A letter was sent to the participants general practice informing them of their patients' participation. On completion of the intervention and where required during the intervention the participants general practice received written communication documenting the vocational advice provided."<br><br>In terms of clinician "buy-in" one of the recruitment methods required a level of engagement with the trial to support recruitment. However, automated methods were developed to support recruitment meaning buy-in was not necessary for the feasibility study. This is documented in the recruitment methods section.
- Intro background: replace "good quality" with "high quality". Also feels like there's a word missing before "active engagement … "	We have amended the word "good" to "high" as requested.<br><br>We have also added the word "supporting" before active engagement to aid clarity.
- Provide more context around fit notes in the UK e.g., are they required? Under what circumstances, etc.	We have updated the section on fit notes in the introduction to include how they are used to manage sickness absence, at what period in the absence and by whom.
- Methods, Design: should be in proper full sentences	We have updated this section for clarity, thank you for highlighting this point.
- Include ethics coverage in methods	In line with the journal requirements, we have included a statement of ethical approval in the declarations section. We can move this if required by the editor.
- Suggest moving description of intervention earlier so it's clear what is being talked about	We have reviewed the methods and moved the description of the intervention to the start of this section as required. Placing it within the other sections would detract from the flow of the methods.
- Add citation for NVivo	We have now added the citation for Nvivo.
- What is the progression criteria? Is this explained somewhere?	We have updated the section on the analysis to indicate that the stop/go criteria were used to make decisions on progression to a full trial. We have also updated the terminology in the discussion section to make it clear that the stop/go criteria were used for this purpose.
- Include when interviews were conducted in relation to surveys and rationale for why only 5 were conducted. Is that representative of the experience?	Page 9 states ", all participants recruited to the feasibility study were invited to participate in a semi-structured interview". We have amended this to include the

	timescale requested which was at any point after the baseline questionnaire was completed.
- Article summary says, “This is the first study to test the feasibility of delivering a VA intervention to patients who present in primary care, regardless of their health condition.” But the study excludes people with serious illness and severe mental health. Contradictory, please clarify.	We have updated this statement to refer to a “range of health conditions” rather than “regardless of health condition” to clarify the point that the intervention did exclude some conditions through necessity.
